# A rare case of ovarian ectopic pregnancy with IUD in situ: A case report from Nepal

**DOI:** 10.1002/ccr3.3393

**Published:** 2020-10-08

**Authors:** Saugat Koirala, Pujan Balla, Ajay Pokhrel, Sachin Koirala, Smriti Pant, Supriya Paudyal

**Affiliations:** ^1^ Department of Obstetrics and Gynecology Dhaulagiri Hospital Baglung Nepal; ^2^ Department of Anesthesia and Critical Care Dhaulagiri Hospital Baglung Nepal; ^3^ Department of Radiodiagnosis and Imaging Dhaulagiri Hospital Baglung Nepal; ^4^ Department of Community Health Sciences Patan Academy of Health Sciences Lalitpur Nepal; ^5^ Department of Emergency Medicine Dhaulagiri Hospital Baglung Nepal

**Keywords:** Copper T, ectopic pregnancy, intrauterine device, IUD, ovarian pregnancy

## Abstract

High index of suspicion of ectopic (much likely ovarian) pregnancy should be considered if a woman with IUD in situ presents with abdominal pain, vaginal bleeding, and positive urine pregnancy test.

## Graphical abstract

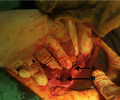

## 1 INTRODUCTION

We present a rare case of ovarian pregnancy in a woman using IUD. IUD leads to mild inflammation of uterus, nearby fallopian tubes, and obstruction in conveyance of ovum. Suspicion of ovarian pregnancy should be considered in a woman using IUD with abdominal pain, vaginal bleeding, and positive pregnancy test.

Copper T is a type of intrauterine device (IUD), which has a contraceptive failure rate of 0.6% with perfect use and 0.8% with typical use.^1^ Several risk factors for ectopic pregnancy have been identified which include previous history of ectopic pregnancy, prior tubal surgery, smoking, and prior IUD use.^2^ Women with history of IUD use in past have 16.27 times more risk of ectopic pregnancy compared with women who have used no contraception.^3^ For every 1000 live births, about 12 visits to the Emergency Department in the United States have been due to ectopic pregnancy.^4^ Ovarian pregnancy, which occurs when the fertilized ovum gets trapped in the ovary, constitutes around three percent of all ectopic pregnancies.^5^ Due to its rare occurrence, there is high chance of ovary being missed as a site of implantation whenever ectopic pregnancy is considered as a differential diagnosis. Although there have been ovarian pregnancies reported from Nepal,^6^, ^7^ to our knowledge this is the first case report of ectopic pregnancy being associated with IUD in situ. With this case report, we want to add to the clinical evidence that relates the concurrent use of IUD with ovarian pregnancy. We are reporting a case of a woman with IUD in situ with ruptured ovarian ectopic pregnancy.

## 2 CASE

A 33‐year‐old lady with G4P3L3 had presented to Emergency Department (ED) of Dhaulagiri Hospital, Nepal, with history of amenorrhea for 43 days and irregular vaginal bleeding for the past 2 weeks with partial soaking of one pad per day. She also had abdominal pain and occasional dizziness on the day she visited the ED. She also gave history of having regular menstruation cycle in the past. Her last child birth was 9 years back. She had been using Copper T (Cu T 380A) as a form of contraception for last 5 years. She gave no history of use of any form of contraception prior to that. She is nonsmoker and gave no history of any medical or surgical illnesses in the past.

**FIGURE 1:**
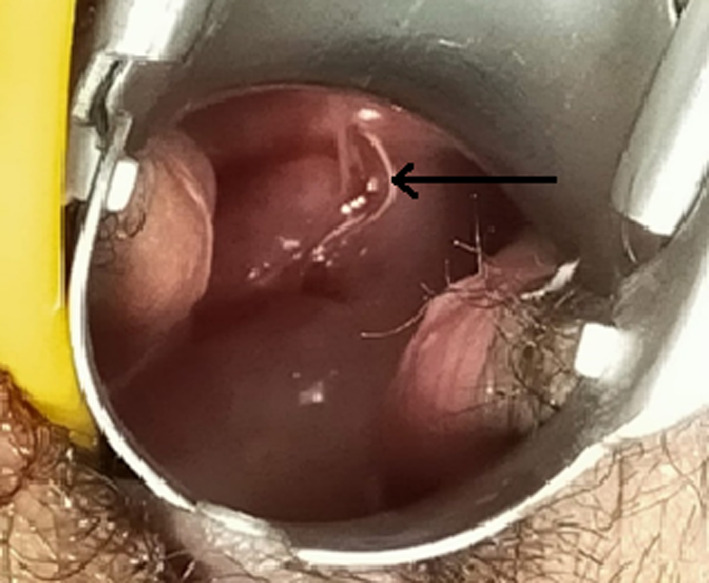
IUD thread visualized in speculum examination as coming from external os. (Arrow: Thread of IUD)

She was well oriented to time, place, and person but looked pale. Her blood pressure was 110 mm Hg systolic and 60 mm Hg diastolic, and pulse was 108 beats per min. On abdominal examination, there was generalized tenderness over the suprapubic region with mild distension. Vulva and vagina looked healthy. Copper T thread was visible on the speculum examination, and cervix looked healthy (Figure 1). Her pelvic examination was significant for a firm mobile mass around the right adnexa of 4 × 4 cm. Despite the history of contraception, the examination findings led to a strong suspicion toward the differential diagnosis of ectopic pregnancy.

**FIGURE 2:**
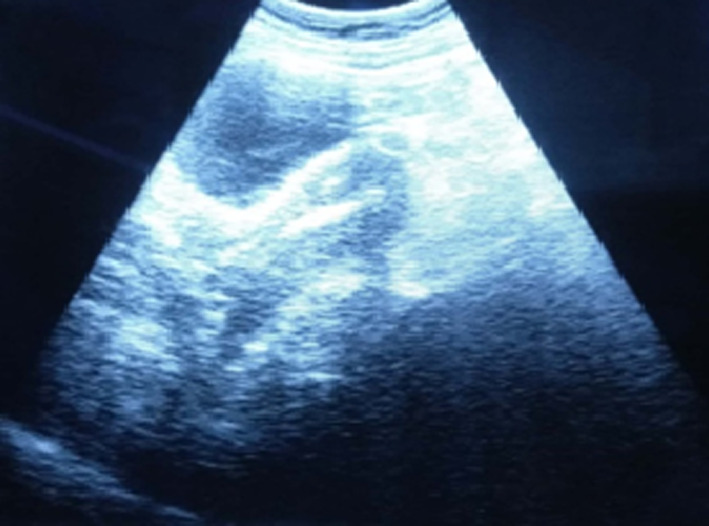
Ultrasonography image showing IUD in situ

We ordered a urine pregnancy test which came out to be positive. We performed an abdominal sonography on her which showed a well‐defined heterogenous lesion of 3.9 × 4.2 cm in the right adnexa overlying the ovary. Ultrasound examination also revealed gross intraperitoneal fluid collection and Copper T in situ (Figure 2).

**FIGURE 3:**
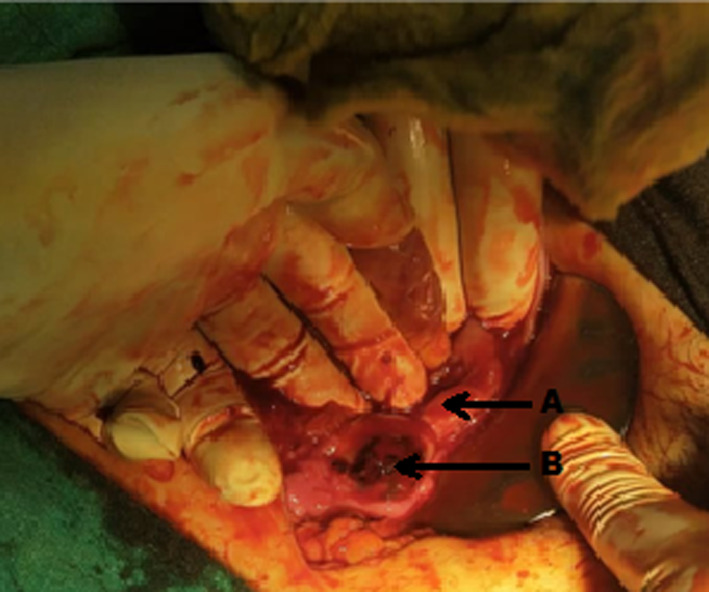
Intraoperative finding of ovarian ectopic pregnancy. (A: Fallopian tube, B: Ruptured ovarian pregnancy)

**FIGURE 4:**
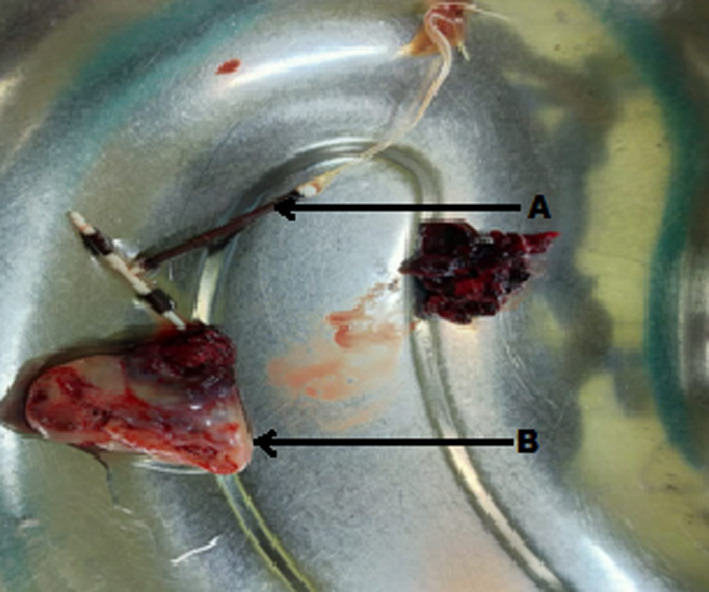
IUD and Resected specimen of ovary. (A: IUD, B: Resected specimen of ovary)

**FIGURE 5:**
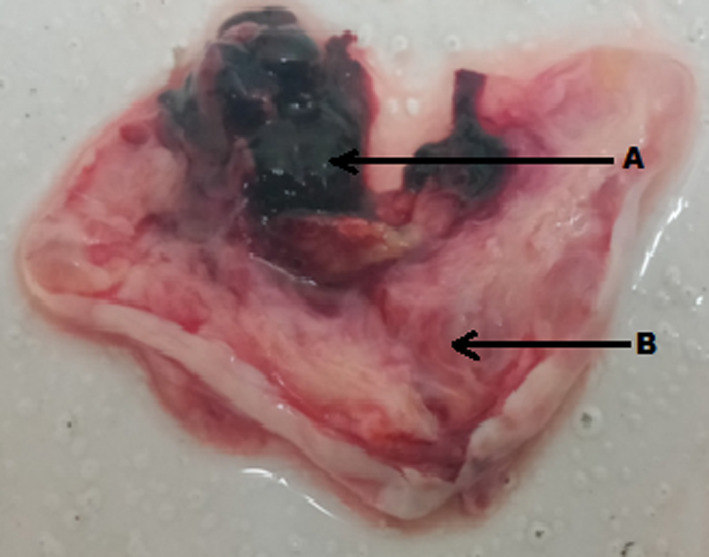
Gross cross section of ovary demonstrating product of conception. (A: Product of conception, B: Ovarian tissue)

We decided to perform exploratory laparotomy on the patient after taking proper informed consent for removal of IUD and the operative procedure. Hemoperitoneum of 1 L was appreciated. The right ovary was 4 × 5 cm in size with a defect of one cm on its surface, a firm mass inside, and clots overlying it (Figure 3). Bilateral fallopian tubes and left‐sided ovary were normal. We performed right‐sided oophorectomy (Figure 4). The IUD string was pulled, and the device was removed in the operation theater (Figure 4).Gross examination of the cross section of the ovary showed products of conception within the ovarian tissue thus confirming ovarian ectopic pregnancy (Figure 5). Her postoperative period was uneventful, and she was pleased with overall management at this center. She gave informed consent for the publication of her case report.

## 3 DISCUSSION

IUD is a top‐tier contraceptive which decreases the incidence of pregnancy overall.^1^ However, various adverse pregnancy events may occur when IUD is in place, namely spontaneous abortion, septic abortion, fetal malformation, preterm labor, and chorioamnionitis.^8^ Women with history of IUD use in the past have significant risk of having ectopic pregnancy compared with women who used no contraception.^3^ Copper T and Levonorgestrel containing IUD are modern generation of IUD with ectopic pregnancy rates of 0.2 per 1000 woman‐year.^9^ Then again, when we consider the possibility of ectopic pregnancy with the current use of IUD, literature has limited answer.

A study, with 287 cases of ectopic pregnancy, done by Pagano had shown 40 patients had IUD in situ.^10^ Among them, 80.5% used Lippes Loop as IUD.^10^ However, given the differences between Lippes Loop and the IUD used at present, it may not be suitable to draw conclusions regarding the relationship between current IUDs and the risk of ectopic pregnancy.

Raziel and colleagues found a strong association of ovarian pregnancy and IUD in situ. Out of 19 diagnosed with ovarian ectopic pregnancy, 13 women (68%) had been using IUD during diagnosis.^11^ But then, this study does not mention the type of IUD or whether it was lying misplaced.^11^

Ercal et al described two cases where ovarian pregnancy was linked with IUD use.^12^ However, both the cases do not qualify for concurrent use of IUD and ovarian pregnancy. While the first case had the IUD removed 1 month prior to the diagnosis of ovarian pregnancy, the other woman had used Lippes Loop for almost 16 years.^12^

Similar to our study, Annaiah and colleagues had reported a case of ovarian ectopic pregnancy with IUD in situ.^13^ In their case, the patient was hemodynamically stable and was managed laparoscopically.^13^ The patient in our study was tachycardic, and ultrasonography revealed features of gross collection intraperitoneally and was managed with laparotomy. Another case of primary ovarian ectopic pregnancy with IUD in situ was managed with laparotomy, wedge reconstruction, and repair of the ovary.^14^ The defect was limited on the superficial surface which was managed with reconstructive procedures in that case. On the contrary, in our case the defect was deep and fetal tissue invaded the ovary. This had prompted for removal of the ovary in our scenario. Findings of tubal ectopic pregnancy with IUD in situ were seen in another case report by Neth et al^15^ Another reported case of ovarian ectopic pregnancy with concurrent use of IUD encourages the use of sonography in women with abdominal pain, vaginal bleeding and positive urine pregnancy tests.^16^ The diagnosis of ectopic pregnancy will not be missed by this approach.

The cellular and humoral components of IUD generate inflammatory reaction of the endometrium. These elements are expressed at the tissue and fluid content of the uterine cavity which prevents fertilization.^17^ The uterine‐tubal junction patency in human allows the passage of air and fluid contents from uterine cavity to the tubal lumen. Thus, the Copper ions in the tube are increased to similar amounts to that in uterine fluid.^18^ This suggests that aside from preventing uterine pregnancy, IUD also protects against tubal ectopic pregnancy. Additionally, in most case studies in women with ectopic pregnancy and IUD in situ are invariably ovarian pregnancies.^11^, ^12^, ^13^, ^14^, ^16^

With these evidences, it looks necessary to further study the association of IUD and ovarian pregnancy and thus highlight the concept of “IUD Associated Ovarian Pregnancy Phenomenon.” The idea is, IUD leads to mild inflammation of the uterus and nearby fallopian tube and also may lead to obstruction in ovum conveyance.^19^ This property of IUD would be responsible for preventive action of it on pregnancy (both on the uterine cavity and the fallopian tube). Additionally, as ovary is devoid of inflammatory action of the device, when ectopic pregnancy occurs, ovary becomes the most likely site of pregnancy.

A long‐term study is required to prove this hypothesis and draw a conclusion to the statement. Nevertheless, limiting factor to conduct this kind of research would be the rarity of pregnancy conceived with IUD and infrequency of ovarian pregnancy overall. Yet, high index of suspicion of ovarian pregnancy should be considered if a woman with IUD in situ presents with abdominal pain, vaginal bleeding, and positive urine pregnancy test.

## CONFLICT OF INTEREST

The authors declare that they have no conflict of interest regarding the publication of this case report.

## AUTHOR CONTRIBUTIONS

SK1, AP, and SP2: involved in diagnosing the patient. SK1, PB, and SK2: involved in treatment and management of the patient (where in SK1 was the leading surgeon; PB and SK2 were the anesthesiologists in charge). SK1 and SP1: wrote majority of the manuscript and formulated the hypothesis. All co‐authors provided critical feedback and helped shape the research, analysis and manuscript.

## ETHICAL APPROVAL

As this was a case report, ethical approval from Institutional Review Board was not sought. However, written informed consent was obtained from the patient.
